# The relationship between interstitial fibrosis and contractile function in HCM: a combined T1-mapping and CSPAMM tagging study

**DOI:** 10.1186/1532-429X-14-S1-O97

**Published:** 2012-02-01

**Authors:** Tevfik F Ismail, Benjamin Hewins, Andrew Jabbour, Niraj Mistry, Pedro F Ferreira, Ankur Gulati, Rick Wage, Patrick Clarysse, Pierre Croisille, Dudley J Pennell, Sanjay K Prasad

**Affiliations:** 1 CMR Unit & NHLI Imperial College London, Royal Brompton Hospital & NHLI Imperial College London, London, UK; 2 Creatis, University of Lyon, Lyon, France

## Background

Interstitial fibrosis is a pathological hallmark of hypertrophic cardiomyopathy (HCM) and is thought to contribute to the abnormal myocardial mechanics seen in this patient group. T1-mapping now allows interstitial fibrosis to be detected non-invasively. Replacement fibrosis as identified by late gadolinium enhancement imaging has been associated with abnormal cardiac mechanics in HCM. However, the relationship between interstitial fibrosis and contractile function in HCM has not been previously explored. We assessed the association between interstitial fibrosis as assessed by T1-mapping and contractile function as determined by continuous spatial modulation of magnetisation (CSPAMM) tagging, hypothesising that increased fibrosis would correlate with abnormal myocardial strain.

## Methods

Twelve HCM patients free of significant comorbidity and 16 controls were studied. CMR was undertaken on a 1.5T Siemens Avanto (Siemens, Erlangen, Germany). Tagged images were acquired at the mid-ventricle using a CSPAMM sequence (Field of View: 300mm, slice thickness 6mm, tag separation 7mm, typical TR/TE 30/1.26ms, Flip Angle 20°, 20 phases). Peak circumferential (Ecc) and radial (Err) strains were determined using inTag (Creatis, Lyon, France). Mid-ventricular short-axis T1 maps were acquired using the Modified Look-Locker Inversion Recovery sequence pre-gadolinium bolus and then at 5, 7, 9, 11, 15, 20 and 25 minutes. Signal intensity-time curves for myocardial and blood pool regions of interest were used to determine T1 relaxation times through a non-linear curve-fit (CMR42, Circle Cardiovascular Imaging, Calgary, Canada). The partition coefficient at equilibrium, an index of fibrosis, was determined by plotting the reciprocal of myocardial T1 times at equilibrium against those for the blood pool and calculating the slope of the resultant linear regression line.

## Results

HCM patients were older than the controls and there was a preponderance of men. In keeping with their diagnosis, HCM patients had significantly higher indexed LV mass and wall thickness than controls (Table 1). The partition coefficient was significantly higher in HCM patients than controls (mean ±SD: 0.439 ±0.230 for HCM vs 0.281 ±0.071 for controls, P=0.02). Whilst peak global Ecc was significantly lower in the HCM group relative to the controls (0.173 ±0.041 vs 0.226 ±0.025, P<0.001), there was no significant difference with respect to peak global Err (0.154 ±0.06 vs 0.134 ±0.051, P=0.25). No significant association was found between the partition coefficient and either circumferential or radial strain.

**Table 1:** 

	Hypertrophic Cardiomyopathy (mean ±SD, n=12)	Hypertrophic Cardiomyopathy (mean ±SD, n=12)	P Value
**Age (years)**	60.0 ±9.67	49.2 ±12.2	0.02
**Male (n, %)**	11	6	0.003
**Indexed LV-EDV (g/m**^2^**)**	77.5 ±9.5	75.8 ±10.1	0.62
**Indexed LV-ESV (g/m**^2^**)**	22.4 ±5.6	23.3 ±5.5	0.66
**LV EF (%)**	71.1 ±6.5	69.4 ±5.4	0.47
**Indexed LV Mass (g/m**^2^**)**	100.9 ±22.8	56.8 ±11.0	<0.001
**Maximum Wall Thickness (mm)**	21.4 ±4.3	7.63 ±1.3	<0.001

## Conclusions

The partition coefficient for gadolinium was significantly raised in patients with HCM relative to controls, however, no association was found between this and local contractile function. This implies that interstitial fibrosis alone may not account for the perturbations in myocardial mechanics seen in patients with HCM and that alternative mechanisms should be explored.

## Funding

This work is supported by the NIHR Cardiovascular Biomedical Research Unit at the Royal Brompton and Harefield NHS Foundation Trust, and Imperial College. Dr Ismail is supported by the British Heart Foundation.

